# Designing Dual-Effect Nanohybrids for Removing Heavy Metals and Different Kinds of Anions from the Natural Water

**DOI:** 10.3390/ma13112524

**Published:** 2020-06-01

**Authors:** Osama Saber, Sarah Mousa Asiri, Mohamed Farouk Ezzeldin, Waleed I. M. El-Azab, Mohammed Abu-Abdeen

**Affiliations:** 1 Department of Physics, College of Science, King Faisal University, P.O. Box 400, Al-Ahsa 31982, Saudi Arabia; 2 Department of Biophysics, Institute for Research and Medical Consultations (IRMC), Imam Abdulrahman Bin Faisal University (IAU), P.O. Box 1982, Dammam 31441, Saudi Arabia; smasiri@iau.edu.sa; 3 Department of Environmental Health, Collage of Public Health, Imam Abdulrahman Bin Faisal University (IAU), P.O. Box 1982, Dammam 31441, Saudi Arabia; mfezzeldin@iau.edu.sa; 4 Egyptian Petroleum Research Institute, Nasr City, P.O. Box 11727, Cairo 11765, Egypt; welazab@yahoo.com; 5 Physics Department, Faculty of Science, Cairo University, Giza 12613, Egypt; mmaabdeen@sci.cu.edu.eg

**Keywords:** adsorption process, dual-function materials, graphene-nanohybrids, porous structures, water quality

## Abstract

In the present study, well-designed nanohybrids are used to act as effective dual-function adsorbents for removing both anions and heavy metals from natural water, at the same time. In this trend, Zn-Al LDHs and graphene oxide are applied to build up building blocks to produce a series of nanohybrids. These nanohybrids were characterized by X-ray diffraction, thermal analyses, Fourier transform infrared spectroscopy, Raman spectroscopy, and scanning and transmission electron microscopy. These techniques confirmed that the prepared nanohybrids contained nanolayered structures with three–dimensional porous systems. These porous systems were identified by the nitrogen adsorption-desorption isotherms and water purification experiments. The obtained results indicated that these nanohybrids included suitable structures to act as dual function materials. The first function was achieved by removing more than 80% of both cadmium and lead from the natural water. The second function was accomplished by eliminating of 100% of hydrogen phosphate and bromide anions alongside with 80%–91% of sulfate, chloride, and fluoride anions. To conclude, these well-designed nanohybrids convert two-dimensional nanolayered structures to three-dimensional porous networks to work as dual-function materials for removing of heavy metals and different kinds of anions naturally found in the fresh tap water sample with no parameters optimization.

## 1. Introduction

It is well known that the quality of drinking water plays an important role in public health of a community. Therefore, the chemical composition of surface water and groundwater, as sources of fresh water supply, is considered a critical issue for water quality [1]. One of the main problems of these water resources is the probability of contamination by several kinds of pollutants such as heavy metals and inorganic anions due to anthropogenic and/or natural activities [2]. The presence of heavy metals in water creates serious human health and environmental issues due to potentially rising toxicity [3]. Lead and cadmium are common examples of heavy metals that threat human health [4]. Concentrations of 1 µg/L of cadmium and 5 µg/L of lead in drinking water could cause fetal diseases like brain and kidney damage to a certain community [5]. The control and removal of excess inorganic anions (such as fluoride, sulfate, hydrogen phosphate, chloride and bromide) in water and wastewater is an essential goal in environmental supervision. The undesirable impacts of these anions, not only on human health and natural ecosystems but also on water network pipelines and treatment facilities over both the short and long term were indicated [6,7,8,9]. Consequently, the development of new techniques to remove heavy metals and inorganic anions from water has become a vital requirement.

Recently, many beneficial processes such as electrocoagulation, sorption and electrochemical separation have been employed to remove several types of pollutants from aqueous mediums [10]. However, many researchers believe that the adsorption process shows satisfactory results in removing of such pollutants because of its low cost, easy operation, and proper efficiency [11]. Among the low-cost adsorbents investigated over the past few years, different kinds of volcanic ash, algae, clay soils, chitin, agricultural wastes, sewage sludge, metallic hydroxides, sludge and industrial wastes were noteworthy [11]. Unfortunately, the majorities of these materials are effective for eliminating only one kind of anion even though some other anions as well as heavy metals might be found in the same matrix. Additionally, these effective adsorbents may not be suitable for all anions because they differ in sizes and valences.

Therefore, the current study focuses on designing dual-function materials to be effective for removing both anions and heavy metals at the same time from real water. These materials were built by combining two building blocks together into well-designed nanostructures. Layered double hydroxide (LDH) and graphene sheets are suitable as building blocks for producing the dual-function nanostructures.

In one hand, LDHs are inorganic layered materials with a general formula of [MII_1−x_MIII_x_(OH)_2_]^x+^(A)^n−^_x/n_·mH_2_O, where MII and MIII are di-valent and tri-valent cations; respectively and A^n−^ is the interlayered anion [12,13]. In the last few decades, LDH markedly received interest for their potential applications in different area, because of their unique properties. High sorption efficiency, low-cost, simple production, and many other benefits make these materials favorable for water purification applications [14]. LDHs materials were also studied as scavengers and sorbents of metal cations from water [15]. On the other hand, graphene (G) and graphene oxide (GO) are one atom-thick carbon layer in a hexagonal lattice in two dimensional spaces with a special kind of compounds and exceptional properties that are technologically interesting [16]. The combination between LDHs and graphene builds up a unique structure of complex composites that takes full benefits of both materials. 

Goh et al. [14] investigated uptake of arsenate As(V) from water by nano-crystalline Mg-Al hydrotalcites with various desorption/sorption conditions. Ni-Al LDH/graphene was prepared by Li et al. [15], for the high adsorption capacities of sodium, arsenite, and arsenate. Gonazlez et al. [17] reported that LDHs show high adsorption capacity to remove copper cations. It was mentioned that LDH/graphene nanocomposites are superior adsorbents for the heavy metals compared with the pristine LDH or graphene [18]. The calcined MgAl-LDH/graphene presented higher adsorption capacity in removing of Cr(VI) from water. Although these various applications of GO-LDHs composites in the field of eliminating of heavy metals, dyes and some organic pollutants from aqueous media were published [10,11,12,13,14,15,16,17,18]. Most of these studies focused only on a single pollutant and are usually found in a synthetic matrix [16]. Consequently, verification of these hybrid materials under natural conditions using real samples that involve multiple elements is widely required.

Therefore, the current study aims to prepare a set of dual-function nanohybrids to remove different kinds of inorganic anions and some heavy metals from natural water of the national network of Eastern providence of Saudi Arabia. These nanohybrids are based on the nanolayered structures of Zn-Al LDHs and graphene oxide. This target can be completed through marriage with two building blocks together into well-designed nanostructures. Certainly, layered double hydroxide (LDH) and graphene sheets are suitable for building blocks to produce the dual-function nanohybrids. 

In this trend, Zn-Al LDHs are prepared and doped by the prepared graphene oxide to build new micro-porous and meso-porous structures through growths of the nanolayers of LDHs on the nanosheets of graphene oxide. In order to maximize the efficiency of these nanohybrids, different percentages of graphene oxide are used to obtain the optimum percentage of graphene oxide for improving the texture of Zn-Al LDHs. Surface properties of the prepared nanohybrids are measured and compared with the pure Zn-Al LDHs. The prepared nanohybrids are also characterized by X-ray diffraction, Fourier Transform Infrared Spectroscopy and Raman Spectroscopy. In addition, a morphological study is investigated through scanning electron microscopy and transmission electron microscopy. The prepared nanohybrids are applied to remove monovalent anions such as: fluoride, chloride, and bromide alongside with di-valent and tri-valent anions such as sulfate and hydrogen phosphate from the natural water. In order to indicate the dual-function effect of the prepared nanohybrids, these materials are applied to remove the heavy metals (cadmium and lead) from the same water sample.

Hence, based to the best knowledge of the authors, the current work would count as a pioneer study for applying nanohybrids as a dual-function adsorbent for removing different kinds of anions as well as cations such as heavy metals in real fresh water.

## 2. Materials and Methods

### 2.1. Preparation of Graphene Sheets

**Figure 1:**
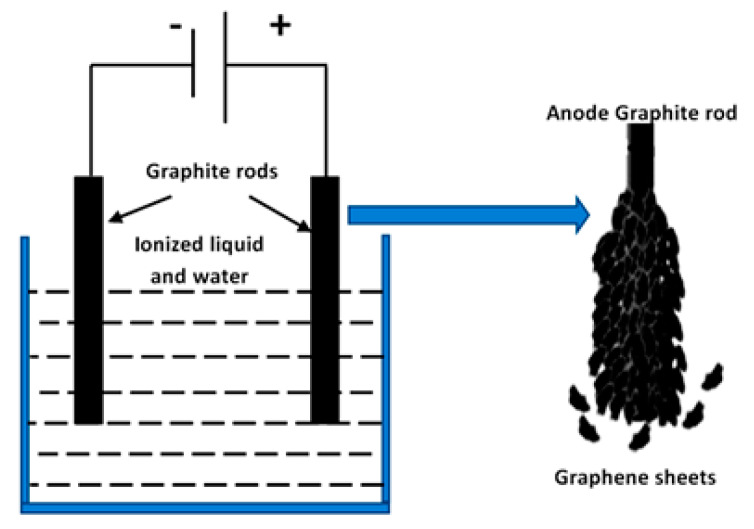
Schematic presentation of the electrochemical cell for producing graphene sheets through the exfoliation process of the graphite anode.

The nanosheets of graphene were prepared by the electrochemical cell as shown in Figure 1. This electrochemical cell used graphite rods as cathode and anode in aqueous solution of imidazolium (ionized liquid) as an electrolyte. During the electrochemical process, water molecules were dissociated and oxidized to produce ·OH radicals in the presence of graphite rods as a catalyst. These radicals were started to bombard the anode by producing graphene sheets which can act as a catalyst. This catalytic effect of the graphene sheet caused simultaneous generation of the hydroxyl radical to accelerate production of graphene sheets through the exfoliation process of graphite [16]. The black product of graphene sheets were separated and washed by ethyl alcohol and then dried at 80 °C.

### 2.2. Preparation of Nanohybrids

In order to avoid hydrophobic behavior of the graphene sheets, they were oxidized through the reaction with hydrogen peroxide in ultrasonic bath. A total of 0.6 g of graphene was suspended in 500 mL of water and 100 mL of hydrogen peroxide (30%) for 2 h. The nanohybrid (NH1) was prepared by the reaction of 0.05 mol of zinc nitrate hexahydrate Zn(NO_3_)_2_∙6H_2_O with 0.02 mol of aluminum nitrate hexahydrate Al(NO_3_)_3_∙9H_2_O in the presence of 0.05 mol of urea CO(NH_2_)_2_. This reaction was achieved in the presence of 0.11 g of the oxidized form of graphene suspended in 750 mL of deionized water. The reaction occurred at 80 °C in the water bath. The grey-white product was precipitated and washed several times by deionized water after 24 h.

Another two nanohybrids were prepared via the same procedure by decreasing the content of graphene oxide. The reaction of zinc nitrate, aluminum nitrate and urea was completed in the presence of 0.07 g and 0.04 g of graphene oxide to produce two new nanohybrids NH2 and NH3; respectively. For comparison, Zn-Al-layered double hydroxide was prepared by the same method without adding graphene oxide.

### 2.3. Characterization

The crystalline structures of Zn-Al LDH and the new nanocomposites were confirmed by X-ray diffraction (XRD–7000, Shimadzu, Kyoto, Japan, with monochromatic high–intensity Cu Kα radiation (λ = 1.5406 Å)) by a scan rate of 0.5°/min. The functional groups were recorded through Fourier Transform Infrared Spectroscopy (FTIR) over the wave number range 400–4000 cm^−1^, using “Spectrum Two” from PerkinElmer Co. The morphology, shape surface and distribution of the samples were obtained directly at room temperature using Scanning Electron Microscope (SEM, Inspect S50, FEI Company, Tokyo, Japan). Transmission Electron Microscopy (TEM, JEM 2100F, JEOL Company, Tokyo, Japan) was carried out at room temperature with an acceleration voltage of 200 kV. Raman spectra measurements were performed using LabRAM HR Evolution, Horiba-Jobin Yvon Technology, with laser 633 ULF. The grating groove density was 300 grooves/mm. The methods of Brunauer–Emmett–Teller (BET) and Barrett–Joyner–Halenda (BJH) were adopted to determine the material surface area, pore volume, and pore size distribution. Inductively coupled plasma emission spectroscopy (ICPE–9800, Shimadzu, Kyoto, Japan) and ion chromatography (930 Compact IC Flex, Metrohm, Riverview, FL, USA) were employed to analyze the concentration of ions in solutions.

### 2.4. Adsorption Experiment

The removal efficiency RE (%) of the samples was investigated at room temperature. Typically, a weight (g) of the sample was stirred in a volume V (L) of fresh tap water (from a national network system) at pH 6 up to 5 h to prepare constant concentrations W/V (10 g/L) of aqueous solutions. In order to study the effect of the adsorbent amount, two other concentrations (20 g/L and 30 g/L) were also used. Before achieving equilibrium, the concentrations of ions in solution were tested every hour. After equilibrium, the suspension was separated by centrifugation, and then the residual concentrations of cations and anions were determined by inductively coupled plasma mission spectroscopy (ICPE) and ion chromatography (IC) as shown in Figure S1. Removal efficiency RE (%) was calculated using the following formula [14].

RE (%) = (C_i_ − C_f_)/C_i_ × 100

where C_i_ and C_f_ (mg/L) is the initial and final concentrations of ions, respectively.

## 3. Results

### 3.1. Powder X-Ray Diffraction

**Figure 2:**
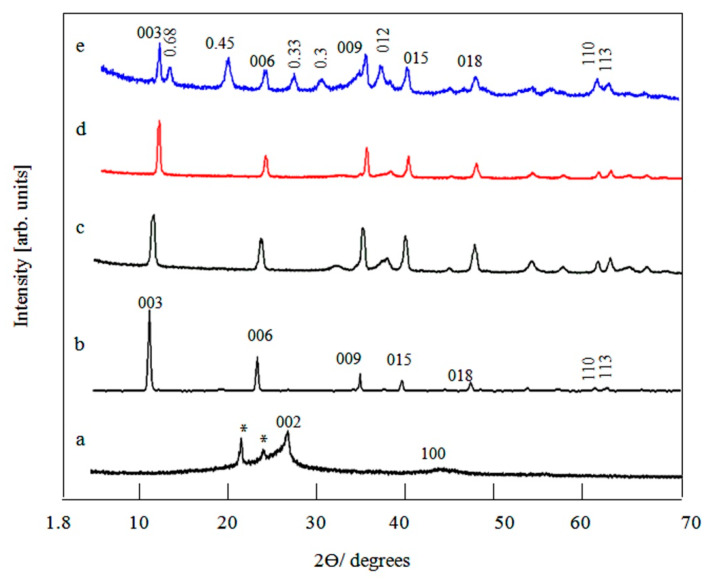
X-ray diffractions of (**a**) the prepared graphene, (**b**) Zn-Al LDH, (**c**) nanohybrid NH1, (**d**) nanohybrid NH2 and (**e**) nanohybrid NH3. * the peaks for the treated graphene.

The crystalline structures of the prepared materials were determined by the powder X-ray diffraction technique as shown in Figure 2. In Figure 2a, the main peak was observed at 2-theta 26.7° which is an indication for the graphene structure. The weak peak, observed at 2-theta 42.9°, confirmed the graphene structure. Both peaks agreed with the reflections of carbon layers in the planes [002] and [100] of graphene. The plane reflection of [002] was used for determining the orientation of the carbon rings in graphene, while, the plane reflection of [100] was due to the condensation degree of the carbon rings in graphene [19,20]. The treated layers of graphene were also confirmed by the appearance of two peaks at 2-theta 21.8° and 24.2° as shown in Figure 2a.

The nanolayered structure of the prepared Zn-Al LDH was confirmed by the X-ray diffraction pattern shown in Figure 2b where, symmetric and sharp peaks were observed at low 2-theta. The peaks aligned with d-spacing of 0.76 nm, 0.38 nm and 0.26 nm. Additionally, asymmetric and weak peaks were detected at high 2-theta which agreed with 0.23 nm, 0.19 nm, 0.17 nm and 0.15 nm. These d-values were due to the planes reflection [003], [006], [009], [012], [015], [110] and [113] of Zn-Al-carbonate LDH (JCPDS file No. 48-1022) [21,22,23,24]. The parameters of the unit cell of the prepared LDH (a) and (c) were calculated by the equations: a = 2 × d-value of plane [110] and c = 3 × d-value of plane [003], respectively. These parameters were 0.310 nm and 2.280 nm similar to our results previously published for LDHs [21].

The X-ray diffraction patterns of the prepared nanohybrids NH1, NH2 and NH3 are displayed in Figure 2c–e. In case of the nanohybrid NH1, clear peaks were observed at 2-theta 11.8, 24.6, 34.7, 37.4, 39.3, 60.3, and 61.6. These peaks were similar to the reflections of the main planes of the prepared Zn-Al LDH [003], [006], [009], [012], [015], [110], and [113]. It means that the nanohybrid NH1 included nanolayered structure whereas the diffraction lines obtained for NH1 were identified as an LDH structure by fitting and matching with the standard entire diffraction pattern of JCPDS 48-1022 as shown in Figure 2c. These diffraction lines exhibited broad peaks. The broadness of diffraction lines showed that the crystallinity of NH1 was in a nanoscale with a layered structure. Although the nanohybrid NH1 contained 3.5% of graphene oxide, the characteristic peaks of graphene oxide were not clear in the pattern. It indicated that the sheets of graphene oxide were completely combined and covered with the structure of LDH.

By decreasing the content of graphene oxide to 2.5%, Figure 2d showed the X-ray diffraction pattern of the nanohybrid NH2. It indicated that the nanohybrid NH2 contained a layered structure with the same characteristic peaks of LDH. However the peaks of graphene oxide were not visile in Figure 1d. It confirmed that graphene oxide sheets were completely dispersed in the matrix of the nanolayered structure of LDH.

Due to continuous reduction of the graphene oxide content to 1.3%, three series of layered structures were observed in the X-ray diffraction of the nanohybrid NH3 as shown in Figure 2e. The d-values 0.750 nm, 0.370 nm and 0.250 nm were found in the first layered structure. The second layered structure included the values 0.680 nm, 0.330 nm and 0.230 nm. These series agreed with the reflections of d003, d006, and d009 and indicated that d003 = 2 × d006 = 3 × d009. The third phase was observed at 0.450 nm and 0.300 nm. These reflections included the planes 006 and 009, which were produced from intercalating the single nanosheets of graphene oxide among the nanolayers of LDHs to appear as a sandwich-type structure of LDHs. These reflections agreed with 2 × d006 = 3 × d009 (2 × 0.450 nm = 3 × 0.300 nm). These results indicated that the nanohybrid NH3 included three different phases of layered structures. It pointed out that the presence of a low percentage of graphene caused growth of different kinds of combinations between the sheets of graphene oxides and the layers of LDH.

The current data represented that graphene oxide played an important role for building up the nanolayered structure of the prepared nanohybrids. The nanohybrids NH1 and NH2 obtained one kind of nanolayered structure while three kinds of nanolayered structures were observed for the nanohybrid NH3. In order to explain this finding, we will discuss the dispersion of the nanosheets of graphene oxide during the growth process of LDH nanolayers. In the case of the nanohybrids NH1 and NH2, the high percentages of graphene oxide exist as groups when building up their nanolayered structures because of their aggregation behavior. It caused formation of one kind of combination between LDH layers with the nanosheets of graphene oxide. For the nanohybrid NH3, the low percentage of graphene oxide gave high dispersion in the medium. Therefore, their nanosheets exist as a single, few and/or groups. During growth of the nanolayers of LDH, three different kinds of interactions occurred with the nanosheets of graphene oxide while producing three different kinds of nanolayered structures. This description was confirmed by analyzing TEM images and surface measurement (will be discussed later).

Another effect was observed for graphene oxide on the crystal size of the LDH layers because of the considerable broadened diffraction peaks of the prepared nanohybrids. Thus, the Debye–Scherer equation was applied to calculate the crystals size of the nanohybrids NH1, NH2 and NH3. According to the peaks of the planes 003, 006 and 009, the mean crystal size of the nanohybrid NH1 was calculated to be 17.0 nm. In case of both NH2 and NH3, they became 26.0 nm and 15.0 nm, respectively. However, the calculations of the pure Zn-Al LDHs showed that its crystal size was 74.40 nm. It highlighted that the presence of graphene sheets played vital role for production platelets of LDHs in the nano scale, which is in agreement with our previous studies for producing nanolayers of LDHs using carbon nanotubes [21].

### 3.2. Scanning Electron Microscopy and Energy-Dispersive X-Ray Spectrometry

**Figure 3:**
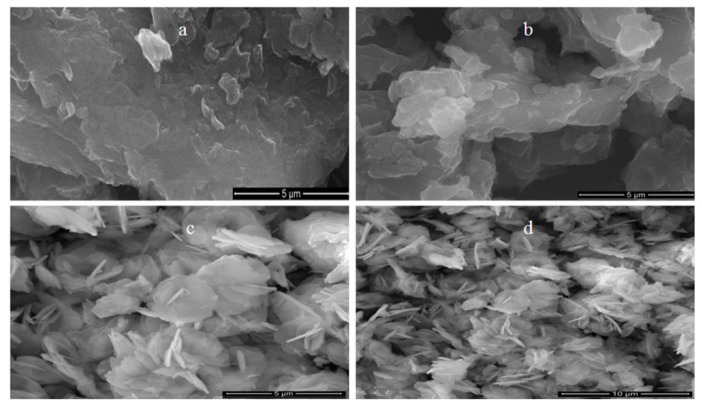
Scanning electron microscopy (SEM) images of (**a**,**b**) prepared graphene and (**c**,**d**) nanohybrid NH1.

It is known that graphene consists of a two-dimensional structure. Therefore, SEM images of the prepared graphene exhibited plate-like morphology as shown in Figure 3a,b. SEM images also revealed strong aggregates for the graphene sheets. This assembling behavior was due to strong intermolecular interactions among the carbon sheets.

SEM images in Figure 3c,d showed the morphology of the nanohybrid NH1. It exhibited individual plates of LDH that included different orientations. This characteristic led to building a porous structure. Although LDH structure is a two-dimensional material, the growth of the LDH layers in presence of graphene oxide converted the nanohybrid NH1 to become a three-dimensional porous structure as shown in Figure 3c,d.

**Figure 4:**
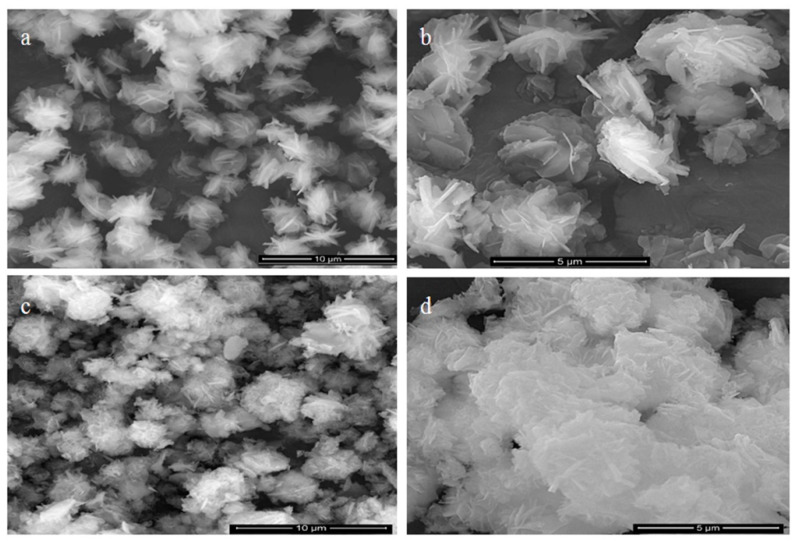
SEM images of (**a**,**b**) nanohybrid NH2 and (**c**,**d**) nanohybrid NH3.

By decreasing the content of graphene oxide, SEM images of the nanohybrid NH2 revealed very small aggregates looking like nanoflowers as shown in Figure 4a. By magnification, Figure 4b showed that these aggregates included cross-linked characteristics of three-dimensional nanoflowers with smooth nanoplates. In case of the nanohybrid NH3, SEM images revealed different types of aggregates, as shown in Figure 4c. These aggregates indicated that there were different kinds of interactions and combinations between layers of LDH and graphene sheets. Additionally, Figure 4d showed that the growth of the nanolayers of LDHs over the graphene nanosheets produced 3D-nanoflowers. 

**Figure 5:**
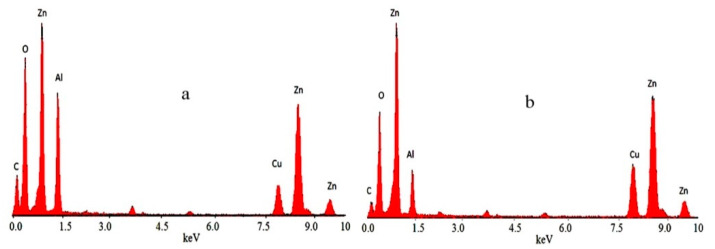
Energy-dispersive X-ray (EDX) spectra of (**a**) nanohybrid NH1 and (**b**) nanohybrid NH3.

Energy-dispersive X-ray spectrometry (EDX) analysis indicated local data of the different elements in the outermost layers of the platelet of the nanohybrids. Zinc, aluminum, carbon and oxygen were clearly identified in the nanohybrids NH1 and NH3. Figure 5 showed that the carbon peak was clearer in the nanohybrid NH1 in comparison to nanohybrid NH3, because of higher content of graphene oxide in the nanohybrid NH1.

### 3.3. Transmission Electron Microscopy

**Figure 6:**
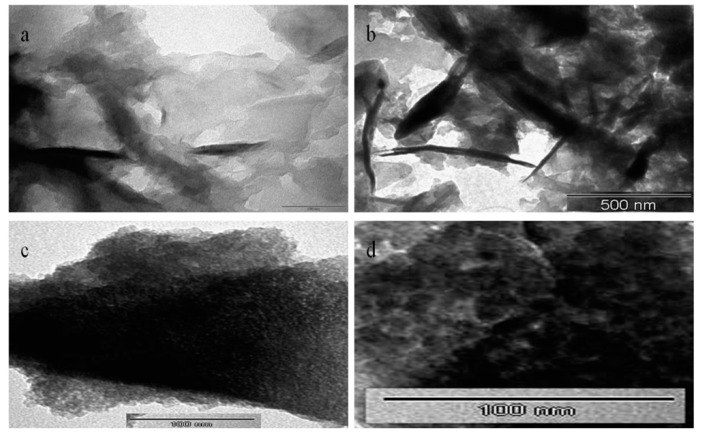
Transmission electron microscopy (TEM) images of (**a**,**b**) prepared graphene and (**c**,**d**) nanohybrid NH1.

It is known that graphene is a single layer of graphite. Consequently, TEM images of the prepared graphene revealed individual and multi-layers in the nano scale as shown in Figure 6a,b. Figure 6a presented large sheets similar to crumpled silk veil, as previously published in the literature [25]. The thin and fine sheets of graphene were observed in Figure 6b.

**Figure 7:**
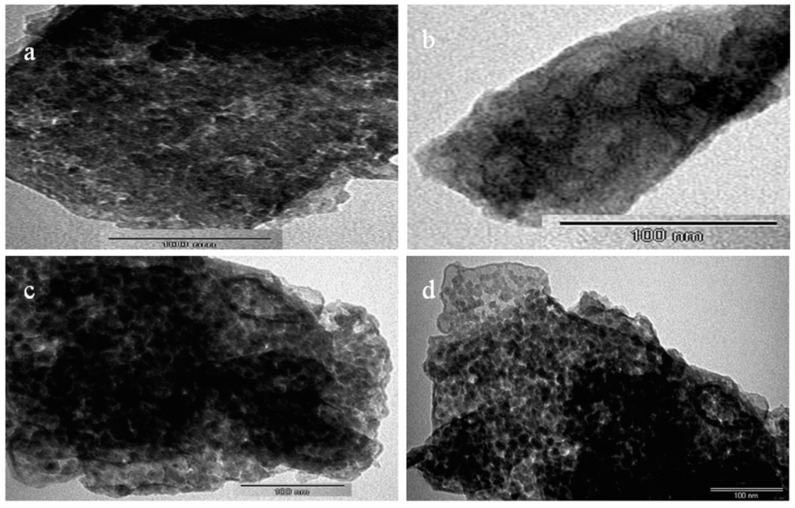
Transmission electron microscopy (TEM) images of (**a**,**b**) nanohybrid NH2 and (**c**,**d**) nanohybrid NH3.

Due to the combination of graphene oxide with LDH, new structures of nanohybrids were released as presented in Figure 6c,d. Figure 6c expressed strong aggregates for the nanohybrid NH1. By magnification, a clear porous structure was observed for the nanohybrid NH1 in Figure 6d. Moreover, transmission electron microscopy (TEM) images of the nanohybrid NH2 revealed the same aggregates as seen in Figure 7a,b. These aggregates were formed by growing the nanolayers of LDH to be curled and perpendicular to the sheets of graphene oxide while producing a 3D-porous structure looking like a nest as shown in Figure 7a. By magnification, TEM images confirmed this explanation because of nanoplatelets of LDH over the graphene sheet that appeared in Figure 6b. In case of the nanohybrid NH3, Figure 7c,d displayed multilayer agglomerates with irregular shapes.

### 3.4. Raman Spectroscopy

**Figure 8:**
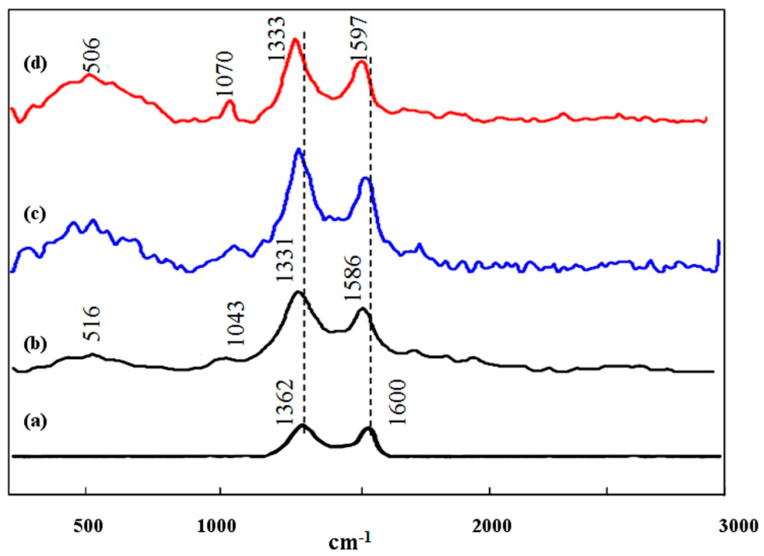
Raman spectra of (**a**) prepared graphene, (**b**) nanohybrid NH1, (**c**) nanohybrid NH2 and (**d**) nanohybrid NH3.

Raman spectroscopy is considered a well-known technique for exploring graphitic materials. Figure 8a presented the Raman spectrum of the prepared graphene. It showed the characteristic bands of graphic materials that were known as D and G bands [26]. The first band was at 1362 cm^−1^ reference to the structural defects and disorder graphitic carbon while, the second band recorded at 1600 cm^−1^ represented order graphitic carbon by confirming the existence of the hybrid sp^2^ atoms of carbon in graphene layers. By forming the nanohybrids based on the prepared graphene oxide, the main bands of graphene oxide were slightly shifted to lower wavenumbers for the nanohybrids NH1, NH2 and NH3. In case of the nanohybrid NH1, Figure 8b showed the main bands at 1331 cm^−1^ and 1586 cm^−1^ by considering the presence of graphene sheets in the matrix of the layered structure of NH1. The observed shift for these bands indicates the strong interaction between the sheets of graphene oxide and the nanolayers of the LDH. New bands were observed at 1043 cm^−1^ and 516 cm^−1^. The new bands were due to M–O–C and M–O–M (M is metal atoms) [27]. It indicated the presence of an interaction between graphene sheets and the layers of LDH. In case of the nanohybrids NH2 and NH3, similar results were observed. The intensity of the D band was higher for the nanohybrids than that of the pure graphene, which confirms the interaction between graphene sheets with an LDH structure [28]. 

### 3.5. Fourier Transform Infrared Spectroscopy

The Fourier Transform infrared (FT-IR) spectroscopy was applied to compare between the function groups of the prepared LDH and the nanohybrids. Table 1 summarized the bands of the functions groups of the LDH and the nanohybrids as well as graphene. The main four function groups of LDH were observed as reported in Table 1, which confirms the presence of OH groups, water molecules and hydrogen bonds alongside with the carbonate anions. Comparing with the structure of LDH, nanohyrids NH1, NH2 and NH3 contained the same function groups that indicated the combination between LDH with graphene oxide did not change their nanolayered structures in agreement with X-ray diffraction results. Additionally, Table 1 confirmed the oxide form of graphene sheets. The carbonate function group was clearly indicated in the spectrum of graphene oxide.

### 3.6. Purification of Water

The concentrations of some selected anions and heavy metals of interest for water quality were analyzed in a fresh sample of tap water from the domestic water network. The results of the analyzed sample were reported in Table 2. 

**Figure 9:**
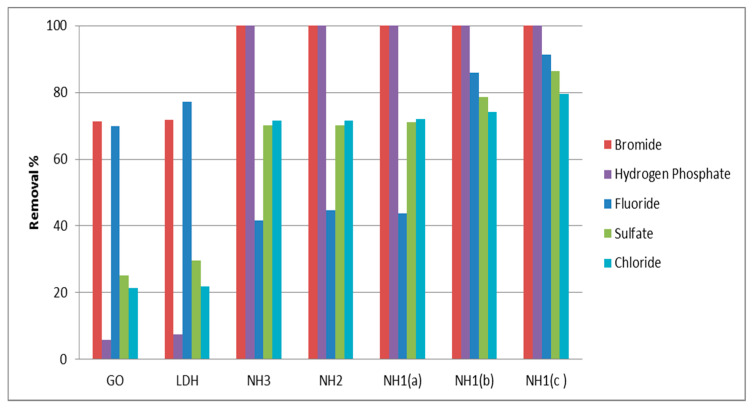
Removal efficiency of selected anions toward the different structures prepared: (**a**) W/V = 10 g/L, (**b**) W/V = 20 g/L, (**c**) W/V = 30 g/L.

The five different materials, previously mentioned, were examined for the water purification experiment GO, LDH, NH3, NH2, and NH1(a) with W/V = 10 g/L, while NH1(b) and NH1(c) were tested with W/V= 20 g/L and 30 g/L; respectively. Additionally, five anions were tested in a water sample for the following adsorption experiments: Br^−^, HPO_4_^2−^, F^−^, Cl^−^ and SO_4_^2−^ as well as two heavy metals, (Cd and Pb). The behavior of these different anions to be adsorbed to the different materials prepared is illustrated in Figure 9. In fact, three different performances were noted. The first behavior was reported for both anions Br^−^ and HPO_4_^2−^. The removal efficiency (RE) of 100% was achieved with all types of nanohybrids NH1, NH2 and NH3. In contrast, adsorption by the parent structures of LDH was reported to be approximately 5% and 70% to HPO_4_^2−^ and Br^−^; respectively. The second attitude was noticed for both SO_4_^2−^ and Cl^−^ ions. Their adsorption was poor (20%–30%) by the parent structure LDH, but gradually improved relative to increasing the amount of GO in structures prepared as well as increasing the W/V factor. However, the maximum RE% for SO_4_^2−^ and Cl^−^ ions was calculated as ~80% for NH1 (W/V = 30 g/L). The obtained results by the current study showed enlargement in the removal percentage of the mentioned anions in comparison to the results concluded by Hatami et al. [29] who tested the removal of some anions from standard aqueous solution using Zn-Al LDH only. They noted that the removal percentage for both hydrogen phosphate and sulfate from model mixed standards was 80% and 20%, respectively, whereas all materials studied did not eliminate chloride from the solution. Some other LDHs with different compositions were only tested for chloride removal by Lv et al. [30] from synthetic aqueous medium. The percentage of removal ranged from 21% up to 64% according to different hybrids applied. These are still below the results of this study. 

Lastly, the RE% for fluoride was 70% and 78% for GO and ZnAl-LDH (pristine structures) respectively, while it achieved up to 91% by the NH1 structure (W/V = 30 g/L). This result is higher than what was reported by Elhalil et al. [31] who mentioned the RE% of 75% for fluoride in real groundwater sample using calcined Mg/Al-LDH. Additionally, the contact time required by the current study for removal of the fluoride ion was 5 h only, whereas Elhalil et al. [31] was 16 h alongside with pH adaptation. Another study for removal of the fluoride ion from aqueous solution was carried out by Kameda et al. [32] who applied Mg-Al-LDH. Kameda et al. were able to remove 60% of fluoride ions prepared in synthetic solution with adjusting parameters such as pH, temperature, etc. However, the results by Zhang et al. [33] indicated that the Li–Al LDHs can be efficiently used to remove fluoride from water with maximum percentage removal (97%) within 1 h. This satisfactory adsorption equilibrium should be combined with adjusting the adsorption conditions as well as using a pure single standard of the fluoride ion, which is not a normal case in the industrial field. 

Hence, one can conclude that occurrence of GO alongside with LDH enhanced the removal efficiency of the tested anions naturally found in tap water. Moreover, it was observed that the modified materials NH1, NH2 and NH3 (same W/V =10 g/L) showed almost the same RE% for all anions studied. However, the nanohybrid NH1 with (W/V = 30 g/L) is highly recommended for the best anion removal from real water samples. 

**Figure 10:**
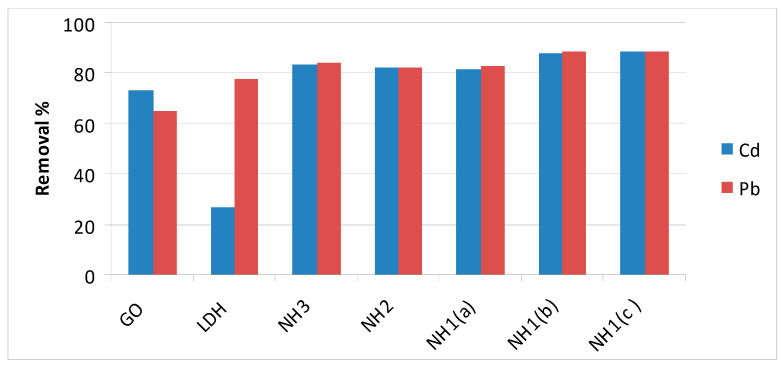
Removal efficiency of selected heavy metals toward the different structures prepared: (**a**) W/V = 10 g/L, (**b**) W/V = 20 g/L, (**c**) W/V = 30 g/L.

The removal efficiency of two important heavy metals related to the water quality issue (Pb & Cd) was tested by applying all structures prepared as shown in Figure 10. The trend of adsorption of both metals toward all synthesized materials was almost the same except for Zn-Al-LDH. RE% for Cd and Pb ions by the pure graphene structure was 73% and 65%, while for Zn-Al-LDH was 26.5% and 77%, respectively. RE% was improved with all the prepared nanohybrids NH1, NH2 and NH3 in comparison to the pristine structures. Additionally, slightly increasing RE was observed according to the order of NH3, NH2 and NH1. The nanohybrid NH1 with (W/V = 30) was highly nominated, once more, for the best Cd and Pb removal (approximately 90%) from real water samples among prepared materials. 

The obtained results are in agreement with both Huang et al. [34] as well as Baruah et al. [35]. Although the current research and these two studies applied GO and LDH by different composition and synthesis methods, the main finding for all, including this work, presented that the removal percentage for Cd and Pb ions in aqueous solution ranged from 90% to 95%. However, it is important to declare that the current work was done on a real fresh water sample without any optimization to experiment conditions. In contrast with using an LDH composite only, the removal percentage for both Cd and Pb ions did not exceed 31% even when experimental conditions (pH, T and contact time) were adapted as reported by Pérez et al. [36]. Hence, one can conclude that occurrence of GO alongside with LDH enhanced the removal efficiency of all tested ions naturally found in tap water. 

To sum up, the present effort would be recognized as a pioneer application for removal of both anions and heavy metals naturally found in a tap water sample by the prepared nanohybrids without experimental optimization. These results concluded that the prepared nanohybrids could work as dual function materials for elimination of different anions from the natural water as well as heavy metals at the same time.

## 4. Conclusions

The current study applied the sheets of graphene oxide and the nanolayers of Zn-Al LDHs to produce well-designed nanohybrids to act as dual-effect adsorbents for removing different anions and two heavy metals from natural water. In this trend, these nanohybrids caused complete removal for sulfate and chloride anions. In addition, they removed the majority of fluoride, bromide and phosphate anions from the natural water. At the same time, these nanohybrids showed another positive effect for removal some heavy metals (cadmium and lead). It confirmed that the prepared nanohybrids can be used as dual-function nanohybrid materials for water purification. This dual-effect of the nanohybrids could be explained by creating new porous structures inside the materials prepared. 

In order to explain the behavior of the prepared nanohybrids toward removal of anions and heavy metals from natural water, the surface area and the porous structure of the nanohybrids NH1, NH2 and NH3 were studied and compared with the parent materials using Brunauer–Emmett–Teller (BET) [37] and Barrett, Joyner, and Halenda (BJH) [38] methods as illustrated in Table 3. The surface properties and porous structures of the prepared materials were determined by full adsorption–desorption isotherm of nitrogen gas at 77 K (−196 °C). A Quanta-chrome Nova sorption system was applied to perform these adsorption–desorption processes. Using the adsorption data, average pore radius, pore volume and specific surface area were assessed through the Brunauer–Emmett–Teller equation. According to the Barrett, Joyner, and Halenda method, the pore size distribution and surface areas were determined by analyzing the desorption data.

The prepared nanohybrids represented high efficiency for removing five anions and two heavy metals even though the sizes of these species are different. This behavior was due to the sharp increasing of the surface area of the nanohybrids in comparison to pure LDH. The surface area of the pure LDH was 5.60 m^2^/g as reported in Table 3. By combining LDH with 3.5% of graphene oxide, the surface area of the nanohybrid NH1 increased to be 33.4 m^2^/g. In the same trend, the surface area of both NH2 and NH3 became 61.50 m^2^/g and 60.3 m^2^/g, respectively. It means that the high efficiency of the nano hybrids can be due to the growth of the surface area. The porous structures of the nanohybrids were better than that of LDH because of new pores with a bigger size being created. The data in Table 3 indicated that the total pore volume of the LDH increased from 0.0120 cm^3^/g to the range of 0.08–0.290 cm^3^/g after combinating with graphene oxide. These pores were more suitable for trapping the different kinds of anions and heavy metals at the same time. These porous structures of the nanohybrids could be obtained by the nanolayers of LDH interacting with the nanosheets of graphene oxide, which act as a substrate for building the nanohybrids. According to our results, we conclude that the advantage of the dual-effect property of these nanohybrids enhanced the removal process of the different kinds of anions and the heavy metals from water in one stage only. It is likely the first time effective materials for eliminating of mono-valent, di-valent, and tri-valent anions were prepared alongside heavy metals in one step.

## Figures and Tables

**Table 1:** Infrared spectra results of the prepared materials.

Assignment	Samples (Wavenumbers, cm^−1^)				
	Zn-Al LDH	NH1	NH2	NH3	Graphene
ν_O–H_	3418	3407	3431	3404	-
Absorption bands (C–H) or H–bond	2988	2986	3048	2915 284	2916 2847
δ_H_2_O_	1649	1606	1637	1637	1698
Absorption bands (COO–)	1429 1360	1355	1360	1434 1355	1562

**Table 2:** Original concentrations of elements of interest for water quality in the analyzed sample.

Anions	mg/L	Heavy Metal	mg/L
Bromide	16.2	Lead	0.25
Hydrogen phosphate	0.35	-	-
Fluoride	0.98	Cadmium	0.23
Chloride	246	-	-
Sulfate	117	-	-

**Table 3:** Surface parameters for the nanohybrids based on Zn-Al layered double hydroxides (LDH) and graphene oxide using Brunauer–Emmett–Teller equation [37] and Barrett, Joyner, and Halenda method [38].

Samples	Graphene Oxide (Wt.%)	S_BET_ (m^2^/g)	S_BJH_ (m^2^/g)	Vp_BJH_ (cm^3^/g)	Rp_BJH_ (nm)
Zn-Al LDH	0.0	5.60	3.30	0.0120	1.80
NH1	3.50	33.40	42.0	0.080	3.50
NH2	2.30	61.50	75.30	0.130	3.20
NH3	1.30	60.30	57.0	0.290	3.50

Rp_BJH_, Vp_BJH_ and S_BJH_, pore radius, total pore volume and specific surface area calculated by the BJH method, respectively; S_BET_, specific surface area calculated by the BET method.

## Supplementary Materials

The following are available online at https://www.mdpi.com/1996-1944/13/11/2524/s1.
