# Supplementary material for: Designing Dual-Effect Nanohybrids for Removing Heavy Metals and Different Kinds of Anions from the Natural Water
Source: Materials (Basel). 2020 Jun 1;13(11):2524. doi: 10.3390/ma13112524 (PMC7321423; doi:10.3390/ma13112524)
Supplement: Supplementary file 1 [file materials-13-02524-s001.pdf]

Instrument Name: ICPE-9800
Model: ICPE-9800
Analyst:

[Analysis Results]

Sample No	7	Weight	1.000000
Sample Classification	UNK	Dilution Rate	1.000000
Sample Name	water	Status Header	Excluded Sample
Date/Time of Analysis	10/26/2017 1:04:15 PM	Status	OFF
Calibration-Curve Group	1 : G1		
Comment			

Quantitive Results

<Intensity>

Element Name	Ca	Cd	Cu	K	Mg	Na	Pb
Wavelength	393.366 (2)	214.438 (2)	327.396 (2)	766.490 (2)	279.553 (2)	588.995 (2)	216.999 (2)
Average	17801.56	80.42253	706.6803	308.7069	4943.365	978.3419	91.82636

<Concentration>

Element Name	Ca	Cd	Cu	K	Mg	Na	Pb
Wavelength	393.366 (2)	214.438 (2)	327.396 (2)	766.490 (2)	279.553 (2)	588.995 (2)	216.999 (2)
Correction							
Unit	ug/L	ug/L	ug/L	ug/L	ug/L	ug/L	ug/L
Average	1270 H	230 L H	21.3 L H	30.6 L H	876 H	42.1 H	247 L H

[Analysis Results]

Sample No	8	Weight	1.000000
Sample Classification	UNK	Dilution Rate	1.000000
Sample Name	osg1-1	Status Header	Excluded Sample
Date/Time of Analysis	10/26/2017 1:11:04 PM	Status	OFF
Calibration-Curve Group	1 : G1		
Comment			

Quantitive Results

<Intensity>

Element Name	Ca	Cd	Cu	K	Mg	Na	Pb
Wavelength	393.366 (2)	214.438 (2)	327.396 (2)	766.490 (2)	279.553 (2)	588.995 (2)	216.999 (2)
Average	2539.001	50.11020	660.0059	304.7904	968.7818	826.7752	58.14550

<Concentration>

Element Name	Ca	Cd	Cu	K	Mg	Na	Pb
Wavelength	393.366 (2)	214.438 (2)	327.396 (2)	766.490 (2)	279.553 (2)	588.995 (2)	216.999 (2)
Correction							
Unit	ug/L	ug/L	ug/L	ug/L	ug/L	ug/L	ug/L
Average	147 H	38.2 L H	16.0 L	27.7 L H	115 H	29.5 L H	39.2 L H

[Analysis Results]

Sample No	9	Weight	1.000000
Sample Classification	UNK	Dilution Rate	1.000000
Sample Name	osg2-1	Status Header	Excluded Sample
Date/Time of Analysis	10/26/2017 1:17:54 PM	Status	OFF
Calibration-Curve Group	1 : G1		
Comment			

Quantitive Results

<Intensity>

Element Name	Ca	Cd	Cu	K	Mg	Na	Pb
Wavelength	393.366 (2)	214.438 (2)	327.396 (2)	766.490 (2)	279.553 (2)	588.995 (2)	216.999 (2)
Average	2794.192	50.57510	681.4960	310.8308	967.8903	861.3843	58.93858

<Concentration>

Element Name	Ca	Cd	Cu	K	Mg	Na	Pb
Wavelength	393.366 (2)	214.438 (2)	327.396 (2)	766.490 (2)	279.553 (2)	588.995 (2)	216.999 (2)
Correction							
Unit	ug/L	ug/L	ug/L	ug/L	ug/L	ug/L	ug/L
Average	166 H	41.2 L H	18.4 L	32.1 L H	115 H	32.4 L H	44.1 L H

[Analysis Results]

Sample No	10	Weight	1.000000
Sample Classification	UNK	Dilution Rate	1.000000
Sample Name	osg3-1	Status Header	Excluded Sample
Date/Time of Analysis	10/26/2017 1:24:43 PM	Status	OFF
Calibration-Curve Group	1 : G1		
Comment			

Quantitive Results

<Intensity>

Element Name	Ca	Cd	Cu	K	Mg	Na	Pb
Wavelength	393.366 (2)	214.438 (2)	327.396 (2)	766.490 (2)	279.553 (2)	588.995 (2)	216.999 (2)
Average	3046.784	50.75036	691.2434	315.9642	1025.619	894.5326	58.63151

<Concentration>

Element Name	Ca	Cd	Cu	K	Mg	Na	Pb
Wavelength	393.366 (2)	214.438 (2)	327.396 (2)	766.490 (2)	279.553 (2)	588.995 (2)	216.999 (2)
Correction							
Unit	ug/L	ug/L	ug/L	ug/L	ug/L	ug/L	ug/L
Average	184 H	42.3 L H	19.5 L	35.8 L H	126 H	35.1 L H	42.2 L H

[Analysis Results]

Sample No	11	Weight	1.000000
Sample Classification	UNK	Dilution Rate	1.000000
Sample Name	osg3-2	Status Header	Excluded Sample
Date/Time of Analysis	10/26/2017 1:31:32 PM	Status	OFF
Calibration-Curve Group	1 : G1		
Comment			

Quantitive Results

<Intensity>

Element Name	Ca	Cd	Cu	K	Mg	Na	Pb
Wavelength	393.366 (2)	214.438 (2)	327.396 (2)	766.490 (2)	279.553 (2)	588.995 (2)	216.999 (2)
Average	1597.309	48.45243	685.0219	320.7024	633.5313	906.5428	56.39593

<Concentration>

Element Name	Ca	Cd	Cu	K	Mg	Na	Pb
Wavelength	393.366 (2)	214.438 (2)	327.396 (2)	766.490 (2)	279.553 (2)	588.995 (2)	216.999 (2)
Correction							
Unit	ug/L	ug/L	ug/L	ug/L	ug/L	ug/L	ug/L
Average	77.7 H	27.8 L H	18.8 L	39.2 L H	50.9 L H	36.1 L H	28.4 L H

[Analysis Results]

Sample No	12	Weight	1.000000
Sample Classification	UNK	Dilution Rate	1.000000
Sample Name	osg3-3	Status Header	Excluded Sample
Date/Time of Analysis	10/26/2017 1:38:22 PM	Status	OFF
Calibration-Curve Group	1 : G1		
Comment			

Quantitive Results

<Intensity>

Element Name	Ca	Cd	Cu	K	Mg	Na	Pb
Wavelength	393.366 (2)	214.438 (2)	327.396 (2)	766.490 (2)	279.553 (2)	588.995 (2)	216.999 (2)
Average	1755.651	48.94470	682.3948	323.0944	658.2667	931.0466	56.57751

<Concentration>

Element Name	Ca	Cd	Cu	K	Mg	Na	Pb
Wavelength	393.366 (2)	214.438 (2)	327.396 (2)	766.490 (2)	279.553 (2)	588.995 (2)	216.999 (2)
Correction							
Unit	ug/L	ug/L	ug/L	ug/L	ug/L	ug/L	ug/L
Average	89.3 H	30.9 L H	18.5 L	41.0 L H	55.6 H	38.2 L H	29.5 L H

[Analysis Results]

Sample No	13	Weight	1.000000
Sample Classification	UNK	Dilution Rate	1.000000
Sample Name	osg4-1	Status Header	Excluded Sample
Date/Time of Analysis	10/26/2017 1:45:11 PM	Status	OFF
Calibration-Curve Group	1 : G1		
Comment			

Quantitive Results

<Intensity>

Element Name	Ca	Cd	Cu	K	Mg	Na	Pb
Wavelength	393.366 (2)	214.438 (2)	327.396 (2)	766.490 (2)	279.553 (2)	588.995 (2)	216.999 (2)
Average	2367.243	53.07578	725.9204	347.9276	837.4436	1016.214	61.23534

<Concentration>

Element Name	Ca	Cd	Cu	K	Mg	Na	Pb
Wavelength	393.366 (2)	214.438 (2)	327.396 (2)	766.490 (2)	279.553 (2)	588.995 (2)	216.999 (2)
Correction							
Unit	ug/L	ug/L	ug/L	ug/L	ug/L	ug/L	ug/L
Average	134 H	57.0 L H	23.5 L H	59.0 L H	89.9 H	45.3 L H	58.3 L H

Sample data

Ident	graphine
Sample type	Sample
Determination start	2018-01-29 13:37:34 UTC+3
Method	An Method Irshad
Operator	

Anions

Data source	Conductivity detector 1 (930 Compact IC Flex 1)	
Channel	Conductivity	
Recording time	35.0	min
Integration	Automatically	
Column type	Metrosep A Supp 5 - 250/4.0	
Eluent composition	Anions - 3.2 mmol/L Na2CO3 + 1.0 mmol/L NaHCO3 in 2 L UPW	
Flow	0.700	mL/min
Maximum flow monitored	yes	
Pressure	10.91	MPa
Maximum pressure monitored	yes	
Temperature	40.0	°C

Anions

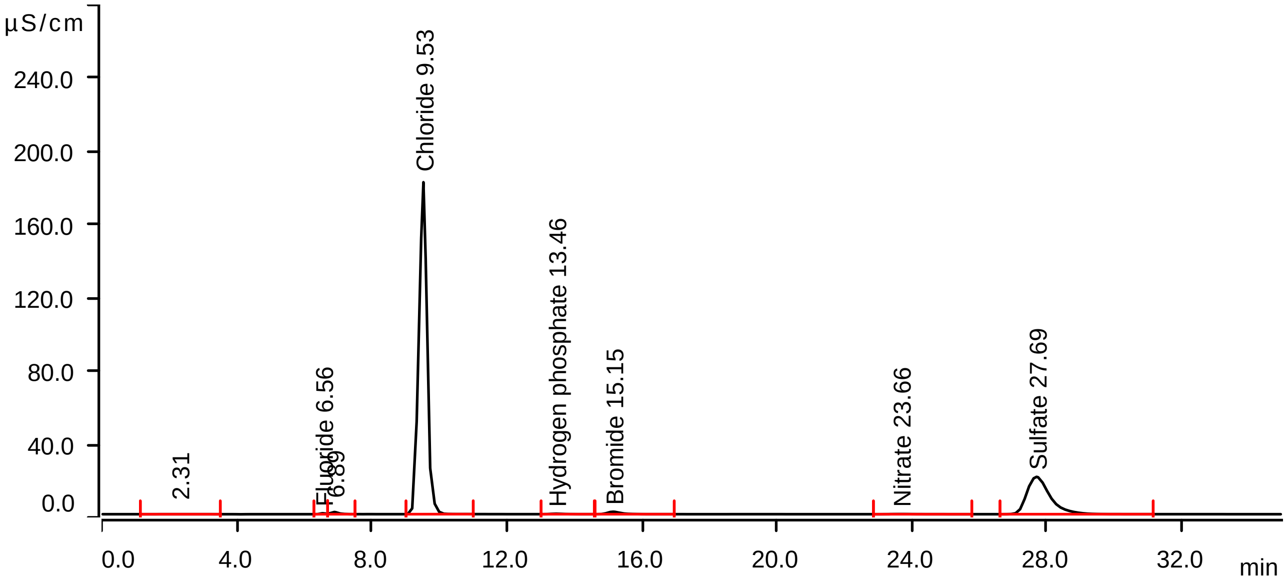

Peak number	Retention time min	Area (µS/cm) x min	Height µS/cm	Concentration ppm	Component name
1	2.305	0.0148	0.014	invalid	
2	6.557	0.0975	0.505	0.297	Fluoride
3	6.893	0.3075	1.185	invalid	
4	9.533	50.3734	181.353	193.493	Chloride
5	13.462	0.1061	0.264	2.151	Hydrogen phosphate
6	15.152	0.6078	1.326	4.665	Bromide
7	23.658	0.0614	0.084	1.157	Nitrate
8	27.688	16.2092	20.413	88.075	Sulfate

Sample data

Ident	HF1 old
Sample type	Sample
Determination start	2018-01-29 14:16:55 UTC+3
Method	An Method Irshad
Operator	

Anions

Data source	Conductivity detector 1 (930 Compact IC Flex 1)	
Channel	Conductivity	
Recording time	35.0	min
Integration	Automatically	
Column type	Metrosep A Supp 5 - 250/4.0	
Eluent composition	Anions - 3.2 mmol/L Na2CO3 + 1.0 mmol/L NaHCO3 in 2 L UPW	
Flow	0.700	mL/min
Maximum flow monitored	yes	
Pressure	10.91	MPa
Maximum pressure monitored	yes	
Temperature	40.0	°C

Anions

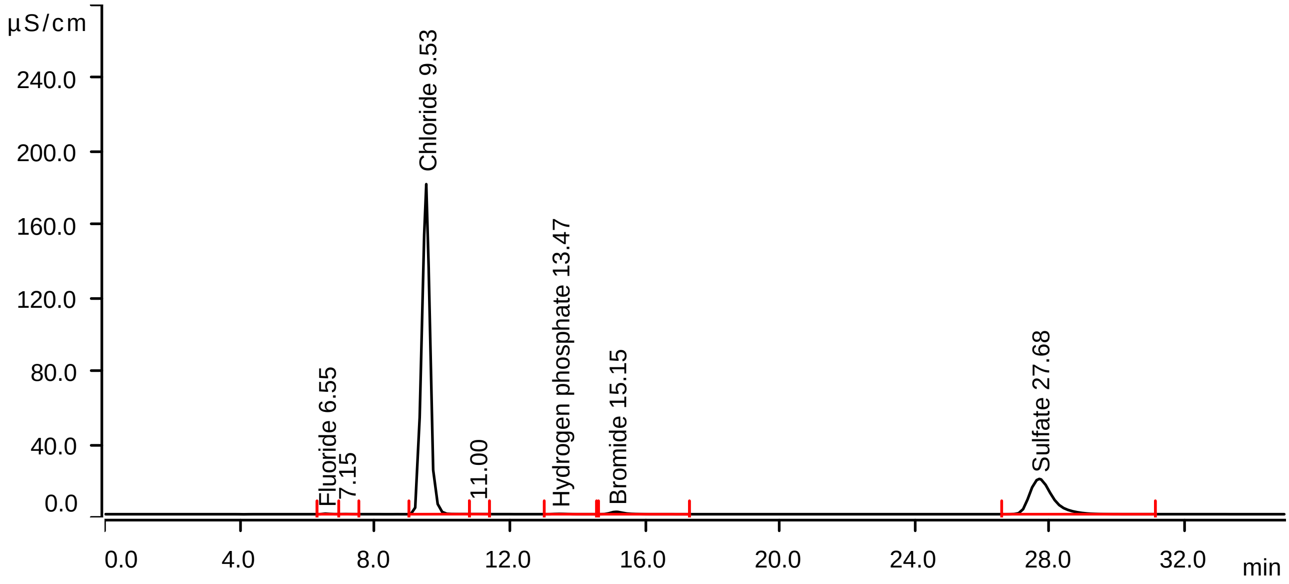

Peak number	Retention time<br>min	Area<br>(µS/cm) x min	Height<br>µS/cm	Concentration<br>ppm	Component name
1	6.552	0.0669	0.304	0.225	Fluoride
2	7.147	0.0172	0.061	invalid	
3	9.528	50.1634	180.287	192.687	Chloride
4	11.003	0.0035	0.012	invalid	
5	13.468	0.0760	0.187	1.750	Hydrogen phosphate
6	15.152	0.5973	1.296	4.592	Bromide
7	27.680	15.2376	19.269	82.811	Sulfate
